# Impact of Hyponatremia and ADH Secretion in MIS-C and COVID-19: An Integrative Approach of Prognostic and Diagnostic Markers

**DOI:** 10.3390/cimb46110698

**Published:** 2024-10-22

**Authors:** Diana-Andreea Ciortea, Carmen Loredana Petrea (Cliveți), Sorin Ion Berbece, Silvia Fotea, Iolanda Cristina Vivisenco, Gabriela Gurău, Mădălina Nicoleta Matei, Aurel Nechita

**Affiliations:** 1 Faculty of Medicine and Pharmacy, University “Dunarea de Jos” of Galati, 800201 Galati, Romania; diana.ciortea@ugal.ro (D.-A.C.); carmen.petrea@ugal.ro (C.L.P.);; 2 Emergency Clinical Hospital for Children “Maria Sklodowska Curie”, 041451 Bucharest, Romania; 3 Emergency Clinical Hospital for Children “Sf. Ioan”, 800487 Galati, Romania; 4 Faculty of Medicine, “Carol Davila” University of Medicine and Pharmacy, 030167 Bucharest, Romania; cristina.vivisenco@umfcd.ro; 5 Department of Pediatrics, Emergency Clinical Hospital for Children “Grigore Alexandrescu”, 011743 Bucharest, Romania

**Keywords:** hyponatremia, ADH, COVID-19, MIS-C, polyuria, SIADH

## Abstract

The COVID-19 pandemic has introduced challenges in pediatric care, especially due to the emergence of Multisystem Inflammatory Syndrome in Children (MIS-C), a severe condition associated with SARS-CoV-2 infection. This study investigated the impact of hyponatremia and antidiuretic hormone (ADH) secretion corelated to clinical outcomes in these patients. We conducted a retrospective cohort study, including 118 pediatric patients, with a detailed sub-cohort analysis of 53 patients for ADH secretion markers. Hyponatremia, defined by age-specific sodium thresholds, was present in 47.22% of MIS-C cases and 28.04% of COVID-19 cases. Ordinal logistic regression analysis revealed that severe hyponatremia significantly increased the likelihood of more severe clinical outcomes (β = 3.514, *p* < 0.001). A significant correlation was found between hyponatremia and prolonged hospitalization. For ADH secretion, a predictive model using ridge regression was analysed, which demonstrated that serum sodium level, U/P ratio, and hospitalization duration are key predictors of SIADH. This model fit was assessed using the ROC curve with an AUC of 0.96, indicating reliable model performance. Our findings underscore the significant role of hyponatremia on the clinical severity and hospitalization outcome of COVID-19 and MIS-C in pediatric patients.

## 1. Introduction

The COVID-19 pandemic has brought different challenges in pediatric care, due to the emergence of Multisystem Inflammatory Syndrome in Children (MIS-C), which is a severe condition linked to SARS-CoV-2 infection. MIS-C is associated with various clinical manifestations, including cardiovascular, gastrointestinal, and renal complications. These pathological aspects, which are also a part of the diagnosis of MIS-C, have significantly increased the complexity of managing infected children. Among these complications, disturbances in sodium balance, particularly hyponatremia, are concerning due to their impact on patient outcomes. Therefore, hyponatremia is a valuable marker that can be used as a predictor of clinical severity and prolonged hospitalization in patients with COVID-19 and MIS-C. Similar to adult studies, this approach based on hydroelectrolitic disturbances has an important impact on the management of pediatric cases [1]. The main pathological directions that directly influence water and electrolyte balance are represented by renal impairment as part of MIS-C or associated with COVID-19, and the effect of antidiuretic hormone (ADH) secretion, which is a critical regulator of water homeostasis, electrolyte balance, and blood pressure.

In COVID-19 pediatric patients, the renal involvement has been well documented and is considered a multifactorial process. Different studies have described acute kidney injury (AKI), nephrotic syndrome, thrombotic microangiopathic damage, acute tubular necrosis, tubular damage, and C3 glomerulopathy [2,3,4,5,6,7]. Renal impairment in MIS-C cases has been less studied. Data reported in the literature to date have identified that AKI is also present in these patients and is associated with a more severe prognosis. The pathogenesis of kidney involvement in MIS-C includes different mechanisms such as: hyperimmune response, inflammatory cytokines, renal hypoperfusion, renin–angiotensin–aldosterone system (RAS) imbalance, endothelial dysfunction, and drug toxicity [8,9]. Based on these pathological findings, the clinical presentation of children with renal impairment and MIS-C is complex, including different elements such as polyuria, hematuria, proteinuria, and pyuria [9,10,11].

Therefore, the diagnosis of renal involvement is also important and has a decisive role in understanding the correct mechanisms of water and electrolytic imbalances in children with COVID-19 and MIS-C. These factors, especially hyponatremia, is of particular concern in this category of pediatric patients because of their strong association with disease severity and prolonged hospital stays.

The other major pathological line in these patients with COVID-19 and MIS-C, that contributes directly to water and hydroelectric balance, is the mechanism of ADH secretion. Osmoreceptors, baroreceptors, and stress are the main mechanisms involved in ADH secretion. Serum osmolality is a direct stimulus for osmoreceptors that release ADH from the posterior pituitary gland. Hypovolemia stimulates baroreceptors from the carotid sinus, carotid body, aorta, and reduced right atrium stretch, which can activate the renin–angiotensin system, causing a baroceptor-mediating mechanism for ADH release, avoiding osmoreceptors [12,13]. In COVID-19 patients, it is well known that produced cytokines, and especially IL6, can cause an inflammatory stress that can affect and influence the non-osmotic ADH secretion, producing the syndrome of inappropriate antidiuretic hormone secretion (SIADH). This pathological mechanism is characterized by excessive secretion of antidiuretic hormone (ADH), which disrupts the normal water and sodium balance, leading to dilutional hyponatremia. Inappropriate ADH activity, exacerbated by cytokine storms associated with COVID-19, plays a crucial role in the pathophysiology of MIS-C and contributes to the complexity of patient care [12,14]. The direct effect of ADH is produced through three types of receptors: V1a, V1b (or V3), and V2, which are heptahelical G protein-coupled receptors with different expression on the cell membrane in different tissues [15]. V2 receptors are mainly expressed in the renal distal tubules and collecting ducts. Through these receptors, ADH stimulates water reabsorption and initially it also stimulates the reabsorption of sodium, leading to increased medullary osmolality and favoring water reabsorption [16]. Through V1 receptors, ADH increases sodium excretion through its influence on blood pressure, effective circulating volume, glomerular filtration rate, and circulation in the vasa recta system [17]. As a result of the SIADH mechanism through these receptors, urinary osmolality usually increases, and diluted serum hyponatremia occurs. One major hallmark of SIADH, which has also been described in patients with MIS-C, is hyponatremia [13,18].

Understanding the complex relationship between ADH secretion, hyponatremia, and clinical severity in pediatric COVID-19 and MIS-C patients offers insight into prognosis and treatment strategies. The present work was conducted based on the interplay of sodium levels, ADH activity, and clinical outcomes observed in a cohort of pediatric patients diagnosed with COVID-19 and MIS-C.

Our study explored and investigated hyponatremia, particularly in the context of inappropriate ADH secretion, as a significant predictor of disease severity and hospitalization outcomes in pediatric COVID-19 and MIS-C patients. The primary outcome of our study was an overview of the relationship between hyponatremia (low sodium levels) and the clinical severity in COVID-19 and MIS-C pediatric patients. Specifically, we aimed to assess how moderate and severe hyponatremia correlates with disease progression, hospitalization duration, and clinical severity stages. Our secondary outcomes included an evaluation of ADH secretion patterns, particularly focusing on the syndrome of inappropriate antidiuretic hormone secretion (SIADH) in these patients. This was investigated through markers such as serum sodium levels and urinary-to-plasma osmolality ratios, which provide insight into the degree of water retention caused by the excessive ADH activity that leads to dilutional hyponatremia and its impact on patient outcomes. Previous studies have not deeply explored these intricate pathological mechanisms in pediatric patients. Moreover, this analysis offers critical insights into ADH secretion pattern and their link to clinical severity and the duration of hospital stay, potentially guiding future treatment strategies and improving patient outcomes.

## 2. Materials and Methods

We conducted a retrospective cohort study with a nested sub-cohort analysis. The study was designed as an exploratory investigation to analyze clinical outcomes, sodium levels, and hyponatremia in the main cohort, which comprised of 118 pediatric patients admitted to different units within the “Sf. Ioan” Emergency Clinical Hospital for children, Galati, Romania. In this cohort, 82 patients were diagnosed with SARS-CoV-2 infection and 36 were cases of Multisystemic Inflammatory Syndrome in Children (MIS-C). Within this main cohort, a nested sub-cohort analysis was performed on 53 patients (36 with MIS-C and 17 with COVID-19) for whom detailed data on urine and serum osmolality were available. This sub-cohort was specifically analyzed to assess ADH secretion and the potential presence of the syndrome of inappropriate antidiuretic hormone secretion (SIADH). All electronic health records were analyzed for relevant demographic data including age and hospitalization duration and the clinical forms were categorized in 5 stages: 1 = asymptomatic, 2 = mild, 3 = moderate, 4 = severe, and 5 = critical. The main laboratory tests of our interest were collected from the medical records of each included patient. This study was conducted in accordance with the ethical standards of the institutional research committee and the Declaration of Helsinki (revised in 2013) and approved by the Medical Board of the Sf. Ioan Children’s Emergency Hospital Galati, under number C399/30 August 2024.

The diagnosis of COVID-19 cases, as well as MIS-C, was based on the clinical definition elements established by World Health Organization (WHO) and by the Center for Disease Control (CDC). Patients were included if they had a confirmed diagnosis of COVID-19 or MIS-C based on the clinical and laboratory criteria outlined in the WHO and CDC guidelines. Diagnosis was based on polymerase chain reaction (PCR) testing for SARS-CoV-2, serological evidence of infection, or clinical features consistent with MIS-C, including fever, elevated inflammatory markers, and multi-organ involvement. Exclusion criteria were established to minimize confounding factors affecting sodium balance or ADH secretion. Patients with pre-existing conditions, such as chronic kidney disease, diabetes insipidus, congenital adrenal hyperplasia, or hypothalamic-pituitary dysfunction, were excluded. Additionally, patients on medications known to influence sodium levels (e.g., diuretics and corticosteroids) or those receiving previous intravenous fluids were excluded from the analysis to ensure accurate sodium assessment.

Children often present with milder symptoms compared to adults, and all the cases included in this study were categorized into one of the five severity groups based on clinical presentation and laboratory findings, as well as radiologic imaging where necessary [19]. The classification into asymptomatic, mild, moderate, severe, and critical illness was conducted at the time of admission, and clinical progression was monitored during the hospitalization period. For COVID-19 patients, the classification was done as it is established by the specific guidelines from the WHO (‘Clinical management of COVID-19: Living guideline’, 18 August 2023) and CDC (‘COVID-19: Clinical care and treatment for children and adolescents, 2023’), which are as follows:

- Asymptomatic infection: children show no symptoms or clinical signs, with a normal radiologic image, in the context of a positive PCR test.
- Mild infection: symptoms of acute upper respiratory infection, such as fever, fatigue, myalgia, cough, dysphagia, rhinorrhea, and sneezing. Physical examination reveals pharyngeal congestion, but no abnormal auscultatory findings. Some cases may present without fever or show digestive symptoms such as nausea, vomiting, abdominal pain, and diarrhea.
- Moderate infection: characterized by pneumonia, and symptoms include fever, and dry cough, followed by productive cough, sometimes with wheezing, but without obvious hypoxemia (normal oxygen saturation > 92%). Some cases may not have clinical signs or symptoms of severe respiratory distress, but chest computed tomography (CT) reveals subclinical lung lesions.
- Severe infection: early respiratory symptoms, such as fever and cough, may be accompanied by gastrointestinal symptoms like diarrhea. The disease typically progresses within about one week, leading to dyspnea with central cyanosis. Arterial blood oxygen saturation is less than 92%. Chest imaging may show bilateral pneumonia.
- Critical infection: children may progress to Acute Respiratory Distress Syndrome (ARDS), septic shock, or multi-organ dysfunction. Symptoms include severe respiratory failure, requiring mechanical ventilation, along with possible encephalopathy, myocardial injury, or heart failure, coagulation dysfunction, and acute kidney injury. Organ dysfunction can be life-threatening.

For MIS-C patients, the severity forms were categorized using 2020 WHO guidelines (‘Multisystem inflammatory syndrome in children and adolescents with COVID-19: Scientific brief’) and the 2023 CDC guidelines (‘Information for healthcare providers about multisystem inflammatory syndrome in children—MIS-C’). Mild MIS-C forms included patients with a fever and mild gastrointestinal symptoms (such as abdominal pain and vomiting) or a rash. Moderate MIS-C implied symptoms such as a persistent fever, a rash, conjunctivitis, hypotension, and moderate cardiac involvement (myocarditis or pericarditis). In this category, two or more organs were affected (e.g., gastrointestinal, cardiac, or respiratory). Severe MIS-C forms consist of multi-organ involvement, including significant cardiac dysfunction (myocarditis or coronary artery aneurysms), shock, or respiratory distress. Children with severe forms often require admission to intensive care, fluid resuscitation, vasopressors, and oxygen or ventilation. Critical MIS-C cases implied progression to life-threatening multi-organ failure, shock, and conditions such as ARDS, acute kidney injury, or encephalopathy.

The paraclinical identification of SARS-CoV-2 was performed using Seegene Nimbus extractor (Seegene Inc., Seoul, Republic of Korea) and CFX 96 amplifier (Bio-Rad Laboratories, Hercules, CA, USA), and the method used was quantitative Reverse Transcription–Polymerase Chain Reaction (qRT-PCR). For the classification of COVID-19 variants in the study population, variant-specific PCR assays were utilized to detect mutations in the spike protein region, allowing for differentiation between the Alpha, Delta, and Omicron variants. Additionally, selected samples underwent sequencing to confirm the variant classifications. The antibodies for establishing and assessing the exact diagnosis in MIS-C cases were identified using YHLO-IFLASH 1800 (YHLO Biotech Co., Ltd., Shenzhen, China) via the chemiluminescence method.

The sodium levels, as well as the rest of the biological parameters used in this study, were measured using the Vitros 4600 chemistry system (Ortho Clinical Diagnostics, Inc., Rochester, NY, USA) and Vitros 5.1FS Chemistry Analyzer (Ortho Clinical Diagnostics, Inc., Rochester, NY, USA), operating with the same technical method. The hyponatremia thresholds considered for our patients respected the recommendations from the producer of these technical systems for each pediatric reference interval as follows (Table 1):

All hyponatremia cases within these intervals were assessed for changes in response to prior administration of intravenous (I.V.) fluids, before the serum determination of sodium, using the Katz and Hiller formulas for potential dysnatremia.

Descriptive statistics were performed, using continuous variables expressed as medians with 25th (Q1)–75th (Q3) percentiles, and categorical variables were reported as frequencies (%).

Inferential statistics were used, such as the Shapiro–Wilk Test, to check data distribution and the Mann–Whitney U test was used to compare continuous variables, in different groups (COVID-19 patients, MIS-C cases, and sodium levels). Fisher’s exact test was applied to compare SIADH indicators between the two sub-groups of the nested sub-cohort for categorial variables.

Given the relatively low incidence of MIS-C and the unique circumstances of the pandemic, the sample size reflected the availability of eligible patients during the study period. To ensure the statistical validity of our results and to verify whether the sample size was sufficient to detect significant differences, we conducted a power analysis for the tests used in the study. The statistical power was calculated using G*Power software version 3.1.9.7 and specific functions from the Python statistical packages (Scipy and Statsmodels).

The Spearman correlation was used to assess the correlation between different variables that were not linearly related and did not require a specific distribution. Also, Kruskal–Wallis tests were used to assess the impact of viral variants in relation to sodium levels, hospitalization duration, and clinical forms. The Kruskal–Wallis test was followed by post-hoc pairwise comparisons represented by the Dunn’s test to determine which groups specifically differed from each other. Odds ratios for the likelihood of hyponatremia across viral variants were not applicable in this analysis due to the nature of the data, but the effect sizes (η^2^) provided a robust measure of the differences. In order to assess the impact of severe hyponatremia on clinical severity an ordinal logistic regression was used to model the probability of a patient having a more severe clinical form of COVID-19 or MIS-C based on the presence of severe hyponatremia.

In the sub-cohort analysis for assessing the ADH secretion and SIADH, descriptive statistics on serum and urinary osmolality, sodium levels, and other relevant markers were performed, as well as a comparative analysis between sub-groups of COVID-19 and MIS-C. For developing a predictive model for SIADH, the multicollinearity was checked using Variance Inflation Factor (VIF) Analysis. Due to the results of VIF values for predictors variables and the small sample size, a penalized regression approach—Ridge Regression (with a L2 penalty)—was chosen, which reduces the effect of multicollinearity by shrinking the coefficients of related variables. This model incorporated the following predictors: serum sodium level (Na), urinary-to-plasma osmolality ratio (U/P Ratio), and the duration of hospitalization. In order to assess the model’s discriminative ability, a Receiver Operating Characteristic (ROC) curve analysis was used. Based on the results, we determined the best threshold for classifying patients as SIADH-positive or SIADH-negative by balancing sensitivity and specificity. All statistical tests were done using Python code, run in IDLE (Python 3.12) environment, with relevant libraries imported, including NumPy 2.1.2 and Pandas 2.2.3 for data manipulation, SciPy 1.14.1 for statistical analysis, and Matplotlib 3.9.2 and Seaborn 3.8+ for data visualization.

## 3. Results

### 3.1. Characteristics of the Two Groups Comprised in the Main Cohort

The main descriptive statistics results were calculated for hospitalization duration, clinical severity, and sodium levels in both groups of patients—those with COVID-19 and the MIS-C group (Table 2). The mean hospitalization duration was significantly higher in the MIS-C group, resulting in 11.00 days (SD 6.81), compared to the COVID-19 group, in which we identified a mean of 5.37 days (SD 5.53). Additionally, the central tendency of the MIS-C group was represented by a median hospitalization duration of 10 days (IQR 7.00–12.00), whereas that of the COVID-19 group was 4 days (IQR 2.00–8.00). The distribution of clinical severity, categorized across the 5 stages, showed that a higher percentage of patients with MIS-C presented with severe and critical clinical illness. Hyponatremia defined for different thresholds for each age interval (<3 years = 135 mmol/L; 3–<6 years = 135 mmol/L; 6–<16 years = 136 mmol/L; and both males and female >16 years = 137 mmol/L), was observed in 28.04% of COVID-19 (n = 23) and 47.22% of MIS-C patients (n = 17).

#### 3.1.1. Assessment of Data Normal Distribution

Before conducting comparative analyses, the normality of the data was assessed using the Shapiro–Wilk test for the following variables: hospitalization duration, clinical severity, and sodium levels in both the COVID-19 and MIS-C groups.

Based on the Shapiro–Wilk test, it was revealed that only the sodium levels in the MIS-C group followed a normal distribution (*p* = 0.82), while all other variables deviated significantly from normality (*p* < 0.001 for all). Given the mixed results, and to maintain consistency in the analysis, a non-parametric test (Mann–Whitney U) was chosen for comparing sodium levels between the groups.

#### 3.1.2. Assessing the Differences in Hyponatremic Children from COVID-19 and MIS-C Groups

When identifying the exact number of cases with hyponatremia in each group, a proportion of 28.04% (n = 23) were diagnosed according to the specific age thresholds (Table 1) in COVID-19 group and 47.22% (n = 17) in the MIS-C group.

In order to assess the differences regarding the exact hyponatremia, the Mann–Whitney test was applied, as shown in Table 3. Both groups were assessed and rank-biserial correlations were calculated to quantify the magnitude of the difference, proving a strong association between the groups (COVID-19 and MIS-C) and hospitalization duration as well as clinical severity, without a significant association with hyponatremia severity.

The test revealed a significant difference in the hospitalization duration between the two groups (U = 110.5, *p* = 0.020), as observed in the general analysis of sodium levels. Children with MIS-C were found to have longer hospital stays compared to the other group. Regarding the clinical severity, there was also a significant difference observed between the two groups (U = 117.5, *p* = 0.026), and it was evident that MIS-C group exhibited more severe clinical forms. These findings were consistent with the general analysis of these clinical outcomes and serum sodium levels, further highlighting the increased severity of MIS-C in hyponatremic patients. The Mann–Whitney U test was also applied to compare the severity of hyponatremia (sodium levels) between the COVID-19 and MIS-C groups. The analysis did not show a statistically significant difference in the severity of hyponatremia between the two groups (U = 231.0, *p* = 0.328).

A post-hoc power analysis was conducted to determine the adequacy of the sample size for detecting significant differences in key outcomes, based on an effect size of 0.8 (considered large for this clinical population), an alpha level of 0.05, and a power of 0.80. The statistical power for the Mann–Whitney U test, used to compare hospitalization duration and clinical severity between COVID-19 and MIS-C patients, was 100% with large effect sizes (0.91 and 1.09, respectively), indicating a high probability of detecting a significant effect between the groups. This demonstrates that the sample size was sufficient to support the conclusions obtained.

**Figure 1:**
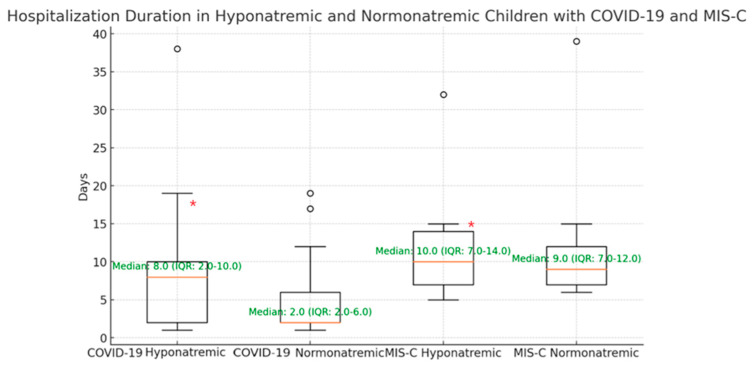
Hospitalization duration in hyponatremic and normonatremic children with COVID-19 and MIS-C. The median hospitalization duration in COVID-19 patients was four times higher than that of the normonatremic children. Regarding the group of MIS-C patients, the difference was much smaller between the two categories of hyponatremic and normonatremic patients. (* indicates *p* < 0.05, statistically significant difference between COVID-19 and MIS-C groups).

In the analysis of hospitalization durations among children with and without hyponatremia in the COVID-19 and MIS-C groups, a detailed visualization assessment was conducted (Figure 1) to determine the distribution of hospital stays across the two groups.

The box plot displays the median (except for the normonatremic COVID-19 group where the median line overlaps with the lower quartile), interquartile range (IQR), and potential outliers that fall outside the whisker range, for hospital stays in each group.

The COVID-19 group showed a more variable distribution of hospitalization duration, especially in those with hyponatremia, having larger whiskers and more outliers. In contrast, the MIS-C group had a more concentrated spread which suggests that there was a smaller variability in hospitalization duration. This visual representation supports the statistical findings that there is a significant difference in hospitalization stay between the two groups in this cohort, with MIS-C patients generally having longer hospital stays, as shown by the position of the median line.

**Figure 2:**
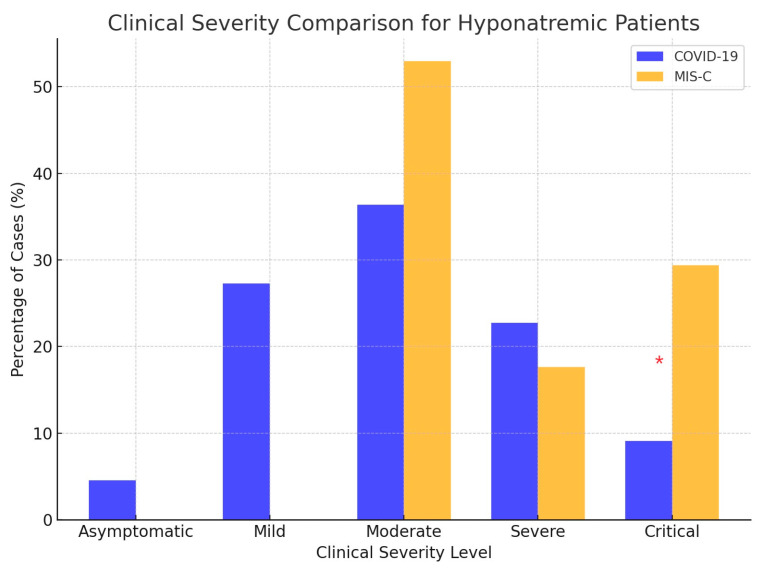
Clinical severity in hyponatremic patients with COVID-19 and MIS-C. The grouped bar chart shows the percentage distribution of clinical severity levels among the hyponatremic children in the COVID-19 and MIS-C groups. Each pair of bars represents the proportion of cases within each clinical severity category (asymptomatic, mild, moderate, severe, and critical) for both groups. (* indicates *p* < 0.05, statistically significant difference between COVID-19 and MIS-C groups).

As shown in Figure 2, the COVID-19 group included significant asymptomatic and mild cases, while the MIS-C group comprised more moderate and critical cases. The bar chart illustrates similar results from the statistical Mann–Whitney test for clinical severity in hyponatremic patients.

#### 3.1.3. General Correlation Between Sodium Levels and Clinical Outcomes

To further examinine the strength and direction of the association between sodium level and clinical severity, as well as the duration of admision into hospital, a Spearman correlation was chosen, especially for the reason that these variables may not have a linear relationship and clinical severity represents an ordinal one. Additionally, there is no normal distribution. The results emphasized that, between sodium levels and clinical severity, there was a negative correlation coefficient (Spearman’s ρ = −0.318, *p* < 0.001), indicating an inverse relationship between these two variables. As sodium levels decrease, the clinical severity tends to increase. The *p*-value was very small, indicating that the correlation was statistically significant. Additionally, there was a significant negative correlation between sodium levels and hospitalization duration (Spearman’s ρ = −0.333, *p* < 0.001), suggesting that patients with lower sodium levels tended to have longer hospital stays.

### 3.2. Analysis of Viral Variants, Clinical Outcomes, and Hyponatremia

This analysis aimed to assess the impact of different viral variants (Alpha, Delta, and Omicron) on sodium levels and clinical outcomes, such as hospitalization duration and clinical forms. Patients infected with the Alpha variant were associated with hyponatremia in 32.79% (95% CI: 23.4–43.1%) of the cases, while the Omicron variant caused hyponatremia in 42.86% (95% CI: 27.9–58.3%) of the cases. The Omicron variant also caused the lowest sodium levels, with a median of 132 mmol/L (95% CI: 130–134) (Table 4).

In order to assess the clinical outcomes of viral variants, a non-parametric method was used, because of the non-normal distribution of the data, as determined by the Shapiro–Wilk test. Therefore, the Kruskal–Wallis test was performed separately for patients with hyponatremia, classified by age-dependent thresholds and those without hyponatremia.

The test indicated significant differences in clinical forms (*p* < 0.001) among viral variants for normonatremic patients, with a large effect size (η^2^ = 0.303), meaning there are substantial differences between viral variants. A post-hoc Dunn’s test revealed significant differences in clinical severity between Alpha and Delta (*p* = 0.007) and Alpha and Omicron (*p* = 0.007). No significant difference was found between Delta and Omicron (*p* = 0.473), regarding the clinical forms. In contrast, when analyzing hyponatremic patients, no significant differences in clinical forms were observed among the viral variants (*p* = 0.95), and the effect size was negligible (η^2^ = −0.052).

For hospitalization duration in hyponatremic patients, the Kruskal–Wallis test revealed no statistically significant difference among patients infected with Alpha, Delta, or Omicron (*p* = 0.60), having a negative value for the effect size (η^2^ = −0.028).

In the second category of non-hyponatremic patients, the results of the Kruskal–Wallis test showed that there was a highly significant difference between the viral variants (*p* < 0.001) in hospitalization duration, showing a large effect size (η^2^ = 0.423). A post-hoc Dunn’s test revealed significant differences in hospitalization duration between Alpha and Delta (*p* = 0.008) and Alpha and Omicron (*p* = 0.008), but not between Delta and Omicron (*p* = 0.473).

**Figure 3:**
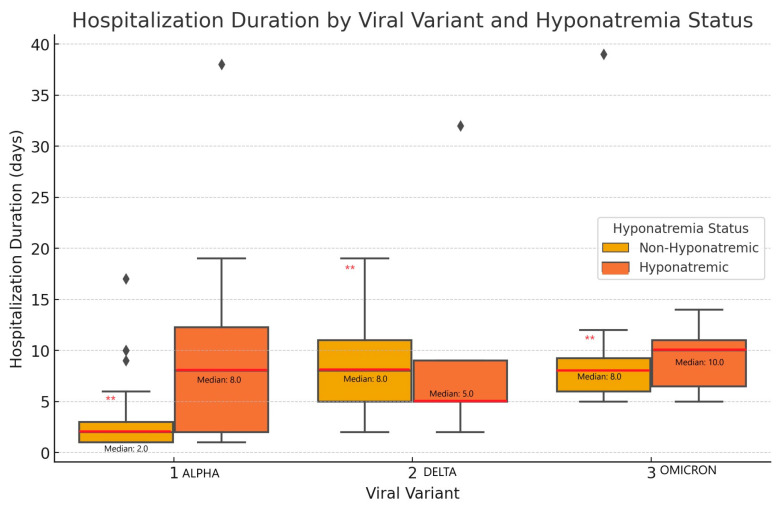
Hospitalization duration by viral variant and hyponatremia status. This figure demonstrates the significant variability of hospital stays across the viral variants in non-hyponatremic patients, with Omicron and Delta variants associated with longer hospitalization duration. (** indicates *p* < 0.001, statistically significant difference between viral variant in non-hyponatremic patients; ♦ represent outliers values; the red and black lines represents the median values).

To illustrate the differences in sodium levels and hospitalization duration, the boxplots in Figure 3 were used. These boxplots show the distribution of the hospitalization duration across viral variants for both hyponatremic and non-hyponatremic patients.

Overall, the presence of hyponatremia was associated with higher mean values in hospitalizations, especially for Omicron and Delta infected patients, in comparison with eunatremic cases (Table 4).

### 3.3. Moderate and Severe Hyponatremia Cases—Distinctive Profiles and Prognoses

For further analysis of hyponatremia impact in children with SARS-CoV-2 infection and MIS-C, the following thresholds were used (Table 5):

The cases that achieved these thresholds varied in age, sodium levels, and hospitalization duration. The clinical form associated with these cases ranged from severe to critical, which indicates the impact of hyponatremia on the clinical presentation.

To explore whether severe hyponatremia correlates with more severe outcomes, a comparative analysis was conducted between these cases and those without hyponatremia, focusing on hospitalization duration and clinical severity.

The mean hospitalization duration in the moderate-to-severe hyponatremia group was 15.8 days, compared to 6.7 days in the non-severe, mild hyponatremia group. The median hospitalization duration was 14.0 days in the moderate–severe hyponatremia group and 6.00 days in the non-severe group.

The Mann–Whitney U test was performed to assess the statistical significance of the differences in hospitalization duration between the two groups. The *p*-value (0.0544) was just above the typical threshold of 0.05, suggesting that the difference between the two groups was marginally non-significant. These results emphasize a trend toward longer hospitalization in moderate and severe hyponatremia groups. However, with a larger sample size of severe hyponatremia cases in COVID-19 and MIS-C pediatric patients, this might reach statistical significance.

**Figure 4:**
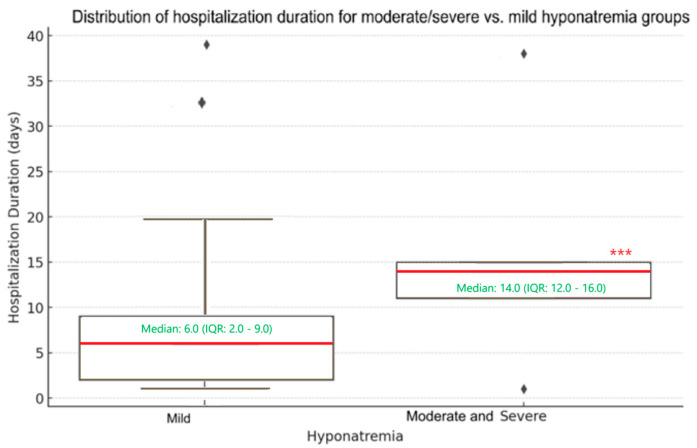
Box-plot for the hospitalization duration distribution for severe versus non-severe hyponatremia groups. The box plot on the left represents the hospitalization duration in the mild hyponatremia group. The box plot on the right shows the hospitalization duration in the moderate and severe hyponatremia groups. (*** represents *p* = 0.0544—indicating a marginally non-significant difference; ♦ represent outliers values; the redlines represents the median values).

For visualizing the results of the comparative analysis, a boxplot has been used (Figure 4). The group of patients with mild hyponatremia had a lower median duration but a broader interquartile range (IQR) and was associated with the distribution of each case, it is evident that it has a greater variability in hospital stays. For the moderate and severe hyponatremia group, the boxplot shows a higher median and a narrower IQR, indicating less variability. Due to the small sample size, in this hyponatremia group, almost all hospital stays were within the IQR, and therefore, there were no other points beyond 1.5 times the IQR, leading to the absence of whiskers. The two extreme values in the hospitalization duration (1 and 38 days), for moderate and severe hyponatremia cases, are considered as part of the data distribution, rather than outliers. Based on the graphical representation, the same trend was observed, indicating that there was a longer hospitalization in the moderate and severe hyponatremia groups.

An ordinal logistic regression was conducted to assess whether severe hyponatremia was a significant predictor of the clinical severity. The analysis yielded the following results, as summarized in Table 6.

The result indicated that severe hyponatremia was a statistically significant predictor of higher clinical severity (coefficient = 3.514, *p* < 0.001). The positive coefficient (β = 3.514) suggests that moderate and severe hyponatremia increase the likelihood of being in a higher category of clinical severity. The confidence interval (95% CI: 1.647 to 5.382) further supports the robustness of this association. The model thresholds (1/2, 2/3, 3/4, 4/5) used are the cut-off points between the ordered categories of clinical severity (1 = asymptomatic, 2 = mild, 3 = moderate, 4 = severe and 5 = critical), reinforcing the impact of severe hyponatremia on clinical prognosis.

The overall significance of the logistic regression model, which included moderate-to-severe hyponatremia as a predictor, was statistically significant, having a Log-Likelihood Ratio Statistic (χ^2^) of 15.20 and a *p*-value of <0.001.

**Figure 5:**
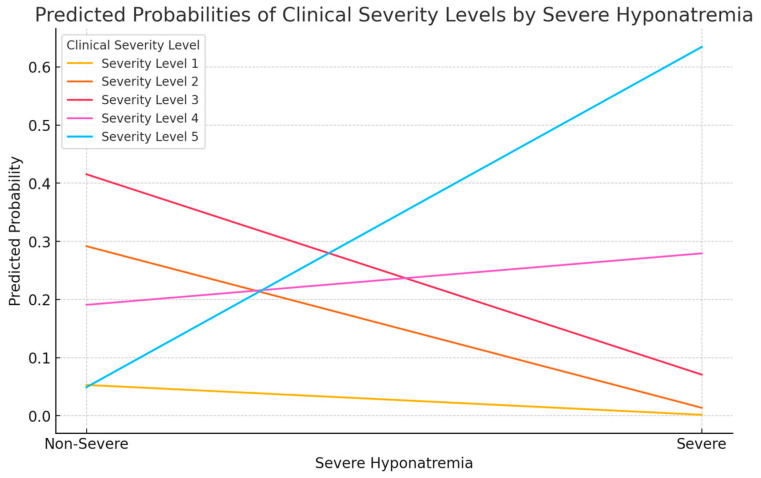
Predicted probability of clinical severity by severe hyponatremia.

In order to emphasize and visually represent the findings from the ordinal logistic regression model, a predictive probability plot was used (Figure 5):

This plot illustrates the predicted probabilities of each clinical severity level (asymptomatic, mild, moderate, severe, and critical) based on the presence or absence of moderate or severe hyponatremia. The x-axis shows the presence (1) or absence (0) of severe hyponatremia. The y-axis indicates the predicted probability of a patient being at each severity level. It displays the likelihood of different severity outcomes across the spectrum of hyponatremia severity and provides a clear visual representation of how severe hyponatremia significantly increases the probability of higher clinical severity.

The plot demonstrates that the probability of being at higher severity levels increases when severe hyponatremia is present. In contrast to this tendency, the likelihood of being in the lower severity categories decreases with severe hyponatremia.

The opposite trend was observed, in which patients without severe hyponatremia had a higher probability of being in less severe clinical categories.

This visualization supports statistical findings and offers a better understanding of the relationship between severe hyponatremia and clinical prognosis.

### 3.4. Nested Sub-Cohort Analysis for ADH Secretion

#### 3.4.1. General Characteristics of ADH Secretion

We analyzed data from 53 patients representing a sub-cohort to assess ADH secretion by examining sodium levels, urinary density, and urinary osmolality, which reflects ADH activity. Blood urea nitrogen (BUN–mg/dL), which indicates the effect of serum levels of urea (mg/dL), and the correction factor (2.14), were used to calculate serum osmolality, associated with serum glucose (mg/dL). The threshold for serum osmolality was <275 mOsm/kg.

For urinary osmolality, the thresholds used vary with age as followed: Infants (up to 1 year): 50 to 600 mOsm/kg; Children (1 year to puberty): 100 to 800 mOsm/kg; and Adolescents (post-puberty): the range is aligned more closely with adult values 300 to 900 mOsm/kg.

In the selected group of 53 patients with MIS-C and COVID-19, the general overview assessment (Table 7), demonstrated the prevalence of high urinary osmolality, which was 30.19%, based on their age-specific thresholds. This result suggests that patients from both subgroups are producing more concentrated urine than expected, which can be indicative of the syndrome of inappropriate antidiuretic hormone secretion (SIADH), especially in the context of hyponatremia. The opposite values for the lower threshold were present in only 6% of the cases, which is less common and could be associated with other alternative mechanisms, such as ADH impairment. The analysis revealed mean Serum Osmolality of 281.33 mOsm/kg and mean Urinary Osmolality of 553.23 mOsm/kg, which were used to calculate the Urine-to-Plasma osmolality ratio for patients with COVID-19 and MIS-C (mean U/P ratio: 1.96).

Assessing the results from each subgroup (Table 8) revealed that urine concentration in the kidneys was higher than expected, especially in the context of low serum osmolality in the MIS-C group. The maximum U/P ratio in the MIS-C group was 5.06, which strongly suggested SIADH. The lower sodium levels in this group suggested that patients might be experiencing dilutional effects due to excess water retention and ADH effects.

#### 3.4.2. Normal Distribution Assessment

All available data were assessed for normal distribution using the Shapiro–Wilks test, which showed that only sodium levels and serum osmolality had normal distribution (*p* = 0.466 and *p* = 0.516 respectively), while sodium osmolality (*p* = 0.003), U/P Ratio (*p* = 0.005), and hospitalization duration (*p* < 0.001) had significantly deviated from normal distribution.

#### 3.4.3. Assessment of Differences in ADH Secretion in COVID-19 and MIS-C Sub-Groups

Taking into account that it is essential to evaluate the differences in ADH secretion indicators between the two sub-groups, and the previous demonstrated differences in distribution for sodium level, serum and urinary osmolality, and U/P ratio, the Mann–Whitney U test was used as it is more suitable for comparing the central tendency (means and medians) of these continuous variables. For categorical variables (the presence of hyponatremia according to the specified age thresholds, high urinary osmolality, and combined SIADH indicators), the Fisher’s exact test was used to compare the proportion of SIADH indicators between the two sub-groups.

The results of the Mann–Whitney U Test (Table 9) from the patients included in this selected group revealed a significant difference in sodium levels between the COVID-19 and MIS-C subgroups. MIS-C patients were likely to have lower sodium levels (*p* = 0.004), which is consistent with hyponatremia. For serum osmolality, the *p* value of 0.020 suggests significant difference between these subgroups, again possibly reflecting the impact of SIADH in MIS-C patients. As assessed in the main cohort of patients when the sodium levels were aliased along with clinical severity and hospitalization duration, in this selected group, the very low *p* value (<0.001) indicates a highly significant difference in hospitalization duration, with MIS-C patients likely having longer stays.

Regarding the categorial variables, the Fisher’s exact test (Table 10) showed a *p*-value of 0.000343 for hyponatremia, suggesting that a highly significant association was present between COVID-19 patients and MIS-C patients that are more likely to present with hyponatremia. These results are concordant with the general finding from the analysis of the main larger cohort. For assessing the presence of high urine osmolality, a *p* value of 0.750 was obtained, which does not represent a significant association between the two pathologies. Although this analysis was performed on a small group of patients, the trend of SIADH indicators, such as the presence of both hyponatremia and high urinary osmolality, should be explored further in a larger sample because, in this case, there was no significant association (*p* = 0.163).

Based on this comparison of the two pathologies, it is evident that hyponatremia and serum osmolality are significantly associated with MIS-C, suggesting that SIADH may be more prevalent in these patients. In addition, as generally observed in the larger cohort, the duration of hospitalization was significantly longer inpatients with MIS-C, and this aspect could be related to the more severe electrolyte disturbances and overall clinical severity. Therefore, these findings provide strong evidence to support the presence of SIADH in the MIS-C group, especially due to the observed differences in sodium levels and serum osmolality.

For the Fisher’s Exact test, used to compare the proportions of hyponatremia between the COVID-19 and MIS-C groups, the power was calculated to be 98.6%, demonstrating that the sample size was adequate to detect differences between the proportions of patients with hyponatremia in the two groups. Although the *p*-value for this test was 0.057, which is close to the significance threshold of 0.05, the results are supported by a very high statistical power.

#### 3.4.4. Correlation Analysis for Assessing the Relationships Between Key Markers of ADH Activity and Clinical Outcomes

Due to the small sample size and the non-normal distribution of some ADH activity markers, the Spearman correlation test was done for different variables. A strong positive correlation (ρ = 0.853, *p* < 0.001) between sodium levels and serum osmolality was demonstrated as both parameters are closely related to the body’s fluid balance and directly correlated to ADH mechanisms of activity.

A moderate negative correlation (ρ = −0.367, *p* = 0.007) was observed, indicating that lower sodium levels are associated with longer hospitalizations duration as seen in the larger cohort. This relationship was statistically significant, suggesting that hyponatremia may be a marker of disease severity and longer recovery times, which could be due to more severe pathological mechanisms such as SIADH.

Regarding the serum sodium levels and urinary osmolality, a weak positive correlation was revealed (ρ = 0.235, *p* = 0.090), but with no statistical significance. Assessing further the correlations between the serum osmolality and urinary osmolality, a Spearman correlation coefficient of 0.241 was obtained, indicating a weak positive correlation, with a *p* value of 0.082, showing no statistical significance.

Additionally, a very weak negative correlation (ρ = −0.051, *p* = 0.719) was proven by the Spearman correlation test for the U/P Ratio and hospitalization duration, suggesting that there is no meaningful relationship between these two variables, and that the ability to concentrate urine relative to plasma osmolality (as reflected in the U/P ratio) does not predict hospitalization stay. In SIADH management, this implies that while U/P ratio can be part of the diagnostic process, it may not be predictive of disease severity or recovery trajectories in the same way that serum sodium levels are.

#### 3.4.5. Predictive Modeling Was Used to Identify the Likelihood of SIADH Based on Serum Sodium, Serum Osmolality, Urinary Osmolality, Hospitalization Duration, and the U/P Ratio

In order to identify the significant predictors of SIADH and to assess the impact of these variables on the likelihood of a patient having SIADH, a thorough assessment was performed. To ensure for the most suitable predictive model, the multicollinearity was checked by calculating the Variance Inflation Factor (VIF), as shown in Table 11.

The high intercept value may be the influence of the small sample size used for this analysis. The high VIF values for Urinary Osmolality and U/P ratio suggested that these variables contribute to multicollinearity and can cause inflated standard errors and *p*-values, making it difficult to determine the individual significance for each predictor used in this model. Therefore, due to the fact that U/P ratio and serum osmolality were highly dependent on one another, the U/P ratio was considered for this predictive model, because it can normalize urine concentration relative to serum concentration, giving a more complex view and be more robust in cases of varying serum osmolality, which is essential in SIADH pathophysiology. As a consequence, a penalized logistic regression—Ridge regression—was used, given the small sample size and multicollinearity. This method is more suitable when dealing with highly corelated predictors, and it can add a penalty to the coefficient to reduce their variance.

All the predictors were standardized to be on the same scale and the logistic regression model used a L2 penalty (Ridge). After implementing these steps, the results were summarized in Table 12.

The results from the Ridge regression model are aligned with the clinical expectations, where lower sodium and osmolality are risk factors for SIADH, higher U/P Ratio represents urinary concentration relative to serum osmolality as a central key for SIADH diagnosis, and longer hospital stays may reflect a more severe form and hydroelectrolitic imbalances. The negative intercept indicates the log-odds of the SIADH when all predictors are at their mean values after standardization. The main predictors assessed, such as sodium, turned a negative coefficient (−0.603), suggesting that lower levels are associated with an increased likelihood of SIADH; serum osmolality also turned a negative coefficient (−0.185), indicating that lower values are also a marker for SIADH, but this effect is smaller than that of sodium. The U/P Ratio had a positive coefficient of 1.135, suggesting that higher values were associated with an increased likelihood of SIADH. This is in accordance with the pathophysiological mechanism as an inappropriate secretion of ADH would lead to a higher urine concentration. And the coefficient for hospitalization duration was positive (0.211), suggesting that longer hospitalization is associated with an increased likelihood of SIADH, indicating that patients with more severe or prolonged illness might be at higher risk, though this effect is small. Overall, the results suggest that the sodium, serum osmolality, and duration of hospitalization are relevant factors

#### 3.4.6. Receiver Operating Characteristic (ROC) Curve Analysis for Assessing Ridge Regression Model Capability to Identify SIADH

To assess the model’s capability to identify SIADH true-positive cases, a Receiver Operating Characteristic (ROC) curve analysis was used.

**Figure 6:**
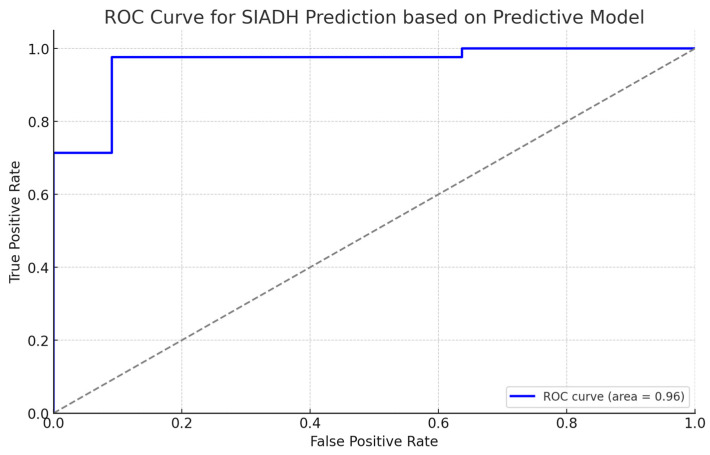
ROC curve for SIADH Prediction Model.

The ROC curve for the predictive model, which included serum sodium levels, U/P Ratio, and duration of hospitalization, demonstrated an Area Under the Curve (AUC) of 0.96 (Figure 6). This indicates excellent model performance in discriminating between patients with and without SIADH. The optimal threshold for classifying patients was 0.61. At this threshold, the model achieved a sensitivity of 97.6% and a specificity corresponding to a false positive rate of 9.1%.

The inclusion of all analyzed predictors associated with the U/P Ratio in the model corresponds to the pathophysiology of SIADH, where inappropriate secretion of ADH leads to higher urine concentration relative to plasma osmolality. The model coefficients indicated that lower serum sodium levels and a higher U/P Ratio were associated with an increased likelihood of SIADH. The ROC analysis confirmed the robustness of the model, using the chosen threshold for a more balanced effect between detecting true cases of SIADH and minimizing false positives.

The ROC curve showed the performance of the Ridge regression model in distinguishing between children with and without SIADH. The model achieved a good performance with an Area Under the Curve (AUC) of 0.96. The threshold value chosen at 0.61 was most optimal in maximizing the model sensitivity at 97.6% while minimizing the false positive rate at 9.1%. This threshold is of great importance in clinical practice, as the early and correct detection of SIADH can make a significant difference regarding the treatment options and prognosis. Given the multicollinearity among predictors, the approach followed was Ridge regression, which increased the stability and reliability of the model.

## 4. Discussion

Our study provides critical insights into the relationship between sodium imbalances, particularly hyponatremia and antidiuretic hormone (ADH) secretion, in pediatric patients with COVID-19 and Multisystem Inflammatory Syndrome in Children (MIS-C). From the main cohort analysis, using the specific age thresholds, it was demonstrated that the prevalence of hyponatremia was significantly higher in the MIS-C group (47.22%) compared to the COVID-19 group (28.04%). This aligns with previous findings in pediatric inflammatory conditions, where hyponatremia is commonly linked to more severe clinical presentations [20,21,22,23,24].

### 4.1. Hyponatremia and Clinical Severity

We identified significant differences in hospitalization duration (U = 110.5, *p* = 0.020) and clinical severity (U = 117.5, *p* = 0.026) between children with COVID-19 and those with MIS-C related to hyponatremia. The results were obtained using a non-parametric method (the Mann–Whitney U test) suitable for non-normal data distribution, as shown by the Shapiro–Wilks test (*p* < 0.001). In addition, the median hospitalization duration was 10 days for MIS-C patients, in contrast to 4 days for COVID-19 patients. This supports the hypothesis that electrolyte disturbances are markers of disease severity, further complicating the management of MIS-C in pediatric care. Additionally, the Spearman’s correlation test demonstrated a statistically significant inverse relationship between sodium levels and clinical severity (ρ = −0.318, *p* < 0.001), as well as between sodium levels and hospitalization duration (ρ = −0.333, *p* < 0.001). These correlations suggested that in both groups, lower sodium levels represented a prognostic marker for severity and clinical outcome.

### 4.2. Sodium Levels and Hospitalization Duration

The analysis of sodium levels, particularly hyponatremia and hospitalization duration confirmed the results from previous analysis [13,25]. As sodium levels decreased, the hospitalization duration increased, indicating that children with more pronounced hyponatremia required longer periods of medical care. Boxplots (Figure 2) were used to emphasize statistical results. It was evident that COVID-19 patients with hyponatremia had more variable outcomes, with some recovering quickly, whereas others stayed in the hospital for significantly longer periods. This variability may be explained by the differences in disease severity, or other aspects, such as underlying conditions or complications related to hyponatremia. For MIS-C patients with hyponatremia, it was shown that hospitalization duration was more consistent and clustered around the median value. This may indicate that MIS-C patients tend to have more uniform clinical courses once hyponatremia is present, with fewer extreme outliers in terms of hospital stay duration. This relationship is critical for understanding the broader clinical impact of sodium imbalances.

### 4.3. Viral Variants and Sodium Levels

The analysis of the influence of identified viral variants from this cohort—namely Alpha, Delta, and Omicron—revealed distinct patterns in hyponatremia and sodium levels. Hyponatremia was more prevalent in patients infected with the Omicron variant (42.86%) compared to the Alpha variant (32.79%). Therefore, from these findings, it seems that different viral variants could influence the body differently as far as sodium balance is concerned, probably because of the inflammatory effect each variant causes systemically. Omicron patients have the lowest median sodium levels (132 mmol/L) and may therefore present a higher risk in terms of sodium imbalance. The lack of significant differences among hyponatremic patients may suggest that sodium imbalance affects clinical outcomes less uniformly across viral variants in contrast to the strong differences observed in normonatremic patients [26].

Hyponatremia is caused, beside the renal impairment and hydro-electrolytic loses, due to the inflammatory mechanisms associated with SARS-CoV-2 infection, leading to the inappropriate secretion of ADH and, hence, SIADH [12,14]. This inappropriate ADH activity can cause water reabsorption, which leads to a secondary dilutional drop in sodium levels [15,16]. Given that Omicron also caused more significant sodium imbalances, further studies should investigate the role of this variant in triggering electrolyte disturbances in pediatric patients.

In order to evaluate the impact of viral variants on hospitalization duration, a non-parametric method was used—the Kruskal–Wallis test—because the data were not normally distributed. Among the hyponatremia group, no difference was seen in hospitalization durations for patients infected with Alpha, Delta, or Omicron (*p* = 0.60). However, in non-hyponatremic patients, the hospitalization duration was significantly longer in those infected with Delta and Omicron compared to Alpha (*p* < 0.001). This finding might suggest that, although hyponatremia itself cannot explain the differences in the duration of hospitalization, some viral variants, including Delta and Omicron, are associated with more severe forms and, respectively, longer recovery times or long-COVID in non-hyponatremic patients [27,28]. This emphasize the importance of viral variant identification, which could predict clinical outcomes, as patients with Delta and Omicron may require longer hospitalization, even if sodium levels are not severely modified. This is of particular interest in pediatric populations, where the correction of electrolyte disturbances is a key approach to improving disease outcomes, and some cases may require more aggressive therapeutic management from the beginning.

### 4.4. Moderate–Severe Hyponatremia: Clinical Outcome

In the present study, we identified the characteristics of pediatric patients who developed moderate and severe hyponatremia defined by specific threshold values of sodium for their age. These patients showed a trend towards longer hospitalization durations compared to the non-severe hyponatremic patients. Although the difference between the two groups was marginally non-significant (*p* = 0.0544), patients with moderate and severe hyponatremia had a mean hospitalization duration of 15.8 days, which was more than double that of the non-severe hyponatremic group (6.7 days). This proves that severe hyponatremia has a bearing on disease progression and recovery time. Therefore, it can be inferred that prolonged duration of stay showed that severe hyponatremia is not only a marker of disease severity, but is also an indicator of long-term recovery [28].

Moreover, it is mentioned in the literature that the association between inflammation and electrolyte disturbances, as seen in MIS-C and severe COVID-19, may exacerbate sodium imbalances through cytokine-mediated mechanisms that contribute to the hyperinflammatory status. These mechanisms, particularly non-osmotic ADH release, lead to water resorption causing a secondary decrease in sodium concentration. Consequently, the clinical course and hospital recovery time will be increased for these high-risk patients [12,13,24].

The outcome of patients with moderate-to-severe hyponatremia was much worse compared to those with mild or no sodium imbalance. An ordinal logistic regression analysis was conducted to assess this, and it was demonstrated that moderate-to-severe hyponatremia was a statistically significant predictor of higher clinical severity (β = 3.514, *p* < 0.001), with patients being more likely to present with severe or critical clinical outcomes. This finding further consolidates hyponatremia as an important marker for disease severity and unfavorable outcomes in COVID-19 and MIS-C cases in pediatric patients.

Syndrome of inappropriate antidiuretic hormone secretion (SIADH) may be main cause of the severe hyponatremia in these cases of COVID-19 and MIS-C patients, as was also described in various other studies for pediatric patients [13,14,29]. In this cohort, the excessive retention of free water due to inappropriate ADH secretion likely contributed to the dilutional hyponatremia observed in these cases.

### 4.5. ADH Secretion and SIADH in MIS-C and COVID-19 Patients

The nested sub-cohort analysis aimed to assess the impact of ADH secretion in the development of hyponatremia and the underlying mechanism of syndrome of inappropriate antidiuretic hormone secretion (SIADH). The overall prevalence of high urinary osmolality was 30.19%, based on age-specific thresholds. This result is an indicator of SIADH in both sub-groups of COVID-19 and MIS-C, because it shows that that these patients produce more concentrated urine than what is normally expected. The analysis revealed that electrolyte disturbances, especially hyponatremia, are more common in critically ill pediatric patients that have systemic inflammatory conditions. Our results indicate that the hyponatremia observed in MIS-C patients (mean value = 134.94 mmol/L) is likely driven by inappropriate ADH secretion, leading to SIADH, as demonstrated by the elevated urinary-to-plasma osmolality ratio (mean value U/P ratio = 1.92) and high urinary osmolality in these patients (mean value = 539.68 mOsm/kg). This finding aligns with previous studies that reported hyponatremia as being related to more severe outcomes in critically ill pediatric patients with MIS-C or COVID [22,23].

The comparison of the two subgroups of COVID-19 and MIS-C using the Mann–Whitney U and Fisher’s exact tests further showed that significant differences were present between sodium levels in both groups (U = 455.0, *p* = 0.004). The patients with MIS-C were more likely to have hyponatremia, lower serum osmolality (U = 429.0, *p* = 0.020) and longer hospitalization stays (U = 16.0, *p* < 0.001), possibly reflecting the impact of SIADH in this sub-group. The results obtained in this study were supported by the adequate statistical power of the tests applied. In the analysis comparing hospitalization duration and clinical severity in COVID-19 and MIS-C patients, the power of the Mann–Whitney U test was 100%, confirming that the sample size was sufficient to detect the observed differences. Additionally, the Fisher’s Exact test, used to compare the proportions of hyponatremia between groups, demonstrated a power of 98.6%, providing high confidence in the validity of the results. Although the *p*-value obtained for this test was not below the significance threshold, the high power suggests that the observed differences may have clinical relevance.

The pathophysiological mechanism underlying SIADH in both COVID-19 and MIS-C seems to be through the inappropriate secretion of ADH, leading to free water retention, which causes dilutional serum hyponatremia and may therefore also contribute to even more severe manifestations in patients [12,13,14,15,16]. This was further evidenced by the strong positive Spearman correlation between serum sodium and serum osmolality (ρ = 0.853, *p* < 0.001) observed in the sub-cohort analysis, indicating that as sodium levels drop, serum osmolality decreases accordingly, which may suggest inappropriate water retention as being the main mechanism. In SIADH, serum osmolality may fail to drop proportionately despite hyponatremia, due to the dilutional effect of excess water [30]. This correlation would indicate that close monitoring of serum sodium and osmolality is very important for the identification and management of SIADH.

The prolonged hospitalization duration observed in MIS-C patients with hyponatremia suggests a link between electrolyte imbalances and recovery time. In the sub-cohort, a moderate negative correlation (ρ = −0.367, *p* = 0.007), was obtained, showing that patients with lower sodium levels have a tendency to stay longer in the hospital, possibly due to the fact that they need intensive monitoring and a correction of electrolyte disturbances. This probably reflects not only the above-mentioned pathophysiological mechanisms but also generally a more severe course of the disease in MIS-C as compared with COVID-19 alone. Early identification of hyponatremia in the disease process may allow for more specific interventions in an attempt to shorten hospitalization and improve patient outcomes.

Although serum sodium and serum osmolality were found to be significantly different between the COVID-19 and MIS-C subgroups, urinary osmolality was not statistically different between the two groups. This finding suggests that while urinary concentration may increase due to ADH activity, it alone may not serve as a reliable marker for distinguishing between the two conditions. This may be due to variations in renal function, fluid administration, or other factors affecting urinary concentration regardless of ADH. Therefore, as previously mentioned in different studies by Bjornstad et al., 2022 and Gambella et al., 2022 [2,5], identifying the correct renal impairment, especially in children with MIS-C, is essential for an accurate diagnosis as well as understanding the underlying mechanism of hyponatremia and water imbalances.

In the management of SIADH, although osmolality is a useful diagnostic criterion, because it typically shows inappropriately high values as a result of excessive ADH secretion, it should not be used as a single diagnostic marker. A comprehensive assessment including serum sodium levels, osmolality, and clinical context is essential for accurate diagnosis and management.

### 4.6. Predictive Modeling and Clinical Implications

These findings have important clinical consequences; thus, hyponatremia may be considered a potential marker of the severity of disease in pediatric COVID-19 and MIS-C patients. Urinary osmolality or U/P ratio alone is unreliable for diagnosis and management in SIADH in view of their poor correlations with clinical outcomes. Therefore, a more complex diagnostic strategy should be used.

For proving this holistic approach, a predictive modeling using Ridge regression was analysed, demonstrating that serum sodium, U/P ratio, and hospitalization duration are key predictors of SIADH. The ROC curve of this model demonstrated an AUC of 0.96, indicating an excellent model performance in discriminating between patients with SIADH and the other cases. The model points out that this is an important indication of the necessity for monitoring these variables in pediatric COVID-19 and, more precisely, in MIS-C patients. Early identification and management of SIADH, if present, by methods such as fluid restriction and close monitoring of sodium levels, may extend better clinical outcomes with a reduced length of hospitalization in MIS-C patients.

Given the strong relationship between sodium imbalances and disease severity in this population, these findings require further confirmation on larger cohorts. Additionally, more studies will be necessary to further explore the molecular mechanisms of ADH dysregulation in MIS-C with a view to developing diagnostic methods and targeted therapies with the capability to correct electrolyte imbalance and improve prognosis in patients.

### 4.7. Limitations

Although the sample size in this study was relatively small, it reflects the challenges in recruiting a larger cohort due to the rare occurrence of MIS-C, especially in pediatric populations. Given the unique nature of this condition, the available cohort during the study period was representative of the cases admitted to our center. In addition, we conducted a post-hoc power analysis confirming the adequacy of our sample size, and it is important to note that MIS-C is a rare condition. One limitation of this study was the relatively small sample size, especially in the sub-cohort analysis of ADH secretion, which can limit the detection of subtle differences between the COVID-19 and MIS-C groups. Additionally, some of these findings need further validation on larger cohorts. Besides, the retrospective nature of this kind of study depends on the available clinical data, which can be incomplete or non-uniform in some cases.

## 5. Conclusions and Future Perspectives

The findings from this study underscore the significant roles that sodium disturbances, particularly hyponatremia, play on the clinical severity and hospitalization outcome of COVID-19 and MIS-C in pediatric patients. The high statistical power of the tests applied supports the robustness of our conclusions, suggesting that the observed differences in clinical severity and hospitalization duration are clinically relevant and may guide future studies and therapeutic interventions in the management of pediatric patients with COVID-19 and MIS-C. Our analysis demonstrated that children with MIS-C were more likely to develop hyponatremia, attributed possibly to inappropriate ADH secretion leading to SIADH. This condition was associated with more severe clinical manifestations and an extended hospital stay.

Moderate and severe hyponatremia in COVID-19 and MIS-C pediatric patients represents an important biomarker that reflects the severity of the disease, prolongs hospitalization, and results in a poor prognosis. Early detection and management could be critical for improving clinical outcomes and reducing the overall burden of care among such patients.

These findings from the sub-cohort analysis further emphasized the pivotal role that ADH secretion plays in the pathogenesis of hyponatremia in pediatric patients with MIS-C. For these cases, a holistic approach that includes serum sodium, serum osmolality, urinary osmolality, hospitalization duration, and U/P Ratio is essential, as demonstrated by the Ridge regression model, verified with the ROC curve results.

Patients showing signs of hyponatremia, especially in the context of MIS-C, should consider the possibility of developing SIADH, because this pathology needs an adapted management for fluid balance. This can include fluid restriction or hypertonic saline administration in severe cases of hyponatremia. Additionally, recognizing the potential role of SIADH in prolonging hospital stays emphasizes the importance of timely diagnosis and intervention.

Furthermore, future research should be done to explore further the molecular mechanisms underlying SIADH in pediatric COVID-19 and MIS-C patients, and targeted therapies should be investigated that can more effectively manage the electrolyte disturbances in these patients. Ideally, larger multicenter cohorts should also be included to confirm these findings and provide more robust evidence.

## Figures and Tables

**Table 1:** Age-specific sodium level reference ranges for pediatric patients.

Age Group	Sodium Level Range (mmol/L)
<3 years	135–142
3–<6 years	135–142
6–<16 years	136–143
>16 years (Females)	137–142
>16 years (Males)	137–143

**Table 2:** Descriptive statistics for hospitalization duration, clinical severity, and sodium levels in pediatric COVID-19 and MIS-C patients.

Variable	Parameter	COVID-19 Group (n = 82)	MIS-C Group (n = 36)
Hospitalization Duration (days)	Mean (SD)	5.37 (5.53)	11.00 (6.81)
	Median (IQR)	4.00 (2.00–8.00)	10.00 (7.00–12.00)
	Minimum duration	1 day	5 days
	Maximum duration	38 days	39 days
Clinical Severity (%)	Asymptomatic	7.32%	-
	Mild	40.24%	-
	Moderate	37.8%	44.44%
	Severe	12.2%	36.11%
	Critical	2.44%	19.44%
Sodium Levels (mmol/L)	Mean (SD)	135.77 (3.84)	134.94 (3.30)
	Median (IQR)	136.00 (135.00–137.00)	135.00 (133.00–137.00)
	Minimum value	116 mmol/L	128 mmol/L
	Maximum value	149 mmol/L	143 mmol/L
Hyponatremia (mmol/L)	Total cases	28.04% (n = 23)	47.22% (n = 17)
<135	<3 years	58.33% (n = 14)	11.76% (n = 2)
<135	3–<6 years	8.33% (n = 2)	17.65% (n = 3)
<136	6–<16 years	16.67% (n = 4)	70.59% (n = 12)
<137	>16 years	16.67% (n = 3)	-
Normonatremic	Total cases	71.95% (n = 59)	52.78% (n = 19)

**Table 3:** Comparison of hospitalization duration and clinical severity in hyponatremic children with COVID 19 and MIS-C.

Variable	U Statistic	Effect Size *	*p*-Value
Hospitalization Duration	110.5	Z = 0.93	0.020
Clinical Severity	117.5	Z = 0.92	0.026
Hyponatremia severity	231.0	Z = 0.84	0.328

* The effect sizes refer to the rank biserial correlation. Children with MIS-C were found to have longer hospital stays and exhibited more severe clinical forms.

**Table 4:** Sodium levels and hospitalization duration by viral variant in pediatric patients with COVID-19 and MIS-C.

Viral Variant	Sample Size (n)	Mean	95% CI (Mean)	Median (IQR)	Standard Deviation
Sodium levels (mmol/L) by viral variant for hyponatremic patients					
Alpha	27	131.70	(129.4–134.0)	133.0 (131.0–135.0)	4.39
Delta	15	134.00	(132.5–135.5)	134.0 (133.0–134.0)	1.22
Omicron	18	132.33	(130.8–133.9)	132.0 (130.0–133.0)	1.95
Hospitalization duration (days) by viral variant for hyponatremic patients					
Alpha	27	9.10	(6.5–11.7)	8.0 (5.0–12.0)	8.72
Delta	15	10.60	(7.5–13.7)	5.0 (3.0–7.0)	12.22
Omicron	18	9.13	(7.5–10.7)	10.0 (8.0–12.5)	3.11
Hospitalization duration (days) by viral variant for normonatremic patients					
Alpha	55	3.17	(2.3–4.0)	2.0 (1.0–4.0)	3.25
Delta	42	8.47	(7.1–9.8)	8.0 (5.0–11.0)	4.39
Omicron	36	9.50	(7.5–11.5)	8.0 (6.0–10.0)	7.28

**Table 5:** Moderate and severe hyponatremia thresholds.

Age Group	Sodium Level for Moderate-Severe Hyponatremia
<3 years	<130 mmol/L
3–<6 years	<130 mmol/L
6–<16 years	<131 mmol/L
>16 years (Females)	<132 mmol/L
>16 years (Males)	<132 mmol/L

**Table 6:** Ordinal Logistic Regression results for moderate–severe hyponatremia as a predictor of clinical severity.

Predictor	Coefficient (β)	Standard Error	z-Value	95% Confidence Interval	*p*-Value
Moderate–Severe Hyponatremia	3.514	0.953	3.689	(1.647, 5.382)	<0.001

**Table 7:** General assessment of the presence of syndrome of inappropriate antidiuretic hormone secretion (SIADH).

Parameter	Mean	Median	Standard Deviation (SD)	1st Quartile (Q1)	3rd Quartile (Q3)	Interquartile Range (IQR)
Serum Osmolality (mOsm/kg)	281.33	281.46	6.81	277.42	285.77	8.35
Urinary Osmolality (mOsm/kg)	553.23	464.28	365.06	249.99	928.56	928.56
U/P Ratio	1.96	1.67	1.29	0.89	3.20	2.31
Duration of Hospitalization (days)	8.40	7.0	6.86	5.0	11.0	6.0

**Table 8:** Assessment of the presence of syndrome of inappropriate antidiuretic hormone secretion (SIADH) in children with COVID-19 and MIS-C.

Parameter	Value—COVID-19	Value—MIS-C
Mean Sodium (mmol/L)	137.59	134.94
Mean Serum Osmolality (mOsm/kg)	284.64	279.77
Mean Urinary Osmolality (mOsm/kg)	581.93	539.68
Mean U/P Ratio	2.04	1.92
Mean Duration of Hospitalization (days)	2.88	11.0
Hyponatremia Prevalence	11.76% (2 out of 17)	44.22% (17 out of 36)
High Urinary Osmolality Prevalence	35.29% (6 out of 17)	27.78% (10 out of 36)
Combined SIADH Indicators	5.88% (1 out of 17)	19.44% (7 out of 36)

**Table 9:** Mann–Whitney U Test—differences in distribution for sodium level, serum and urinary osmolality, and U/P ratio.

Variable	U Statistic	Effect Size (r)	*p*-Value	Significance
Na (mmol/L)	455.0	0.28	0.004	*
Serum Osmolality	429.0	0.21	0.020	*
Urinary Osmolality	327.5	0.05	0.689	No significance
U/P Ratio	324.0	0.03	0.739	No significance
Duration of Hospitalization	16.0	0.55	<0.001	**

* for *p* < 0.05 and ** for *p* < 0.001.

**Table 10:** Fisher’s Exact Test—comparison of the proportion of syndrome of inappropriate antidiuretic hormone secretion (SIADH) indicators between COVID-19 and MIS-C sub-groups.

Variable	U Statistic	Effect Size (Phi)	*p*-Value	Significance
Hyponatremia	0.000	0.45	0.000343	**
High Urinary Osmolality	1.418	0.05	0.750	No significance
SIADH Indicators	0.000	0.18	0.163	No significance

** for *p* < 0.001.

**Table 11:** Variance Inflation Factor for syndrome of inappropriate antidiuretic hormone secretion (SIADH) predictors.

Variable	Variance Inflation Factor (VIF)	Multicollinearity
const (Intercept)	5608.89	very high
Na (mmol/L)	5.32	moderate
Serum Osmolality	6.73	moderate
Urinary Osmolality	1839.64	high
U/P Ratio	1811.35	high
Duration of Hospitalization	1.10	low

**Table 12:** Ridge regression for predicting syndrome of inappropriate antidiuretic hormone secretion (SIADH).

Variable	Coefficient
Intercept	−2.336
Na (mmol/L)	−0.603
Serum Osmolality	−0.185
U/P Ratio	1.136
Duration of Hospitalization	0.211

## Data Availability

Personal medical data are publicly unavailable due to privacy or ethical restrictions as the data were obtained from the medical records of patients admitted to the Emergency Clinical Hospital for Children Sf. Ioan Galati.

## References

[1] Tzoulis P. (2021). Prevalence, Prognostic Value, Pathophysiology, and Management of Hyponatraemia in Children and Adolescents with COVID-19. Acta Biomed..

[2] Gambella A., Barreca A., Biancone L., Roccatello D., Peruzzi L., Besso L., Licata C., Attanasio A., Papotti M., Cassoni P. (2022). Spectrum of Kidney Injury Following COVID-19 Disease: Renal Biopsy Findings in a Single Italian Pathology Service. Biomolecules.

[3] Taş N., Uslu Gökçeoğlu A., Aykaç K., Cura Yayla B.C., Şeneş M., Demirkapı L., Çolak Samsun E. (2022). Evaluation of Tubular Dysfunction Using Urine Biomarkers in Children with COVID-19. Turk. Arch. Pediatr..

[4] Devrim F., Böncüoğlu E., Kıymet E., Şahinkaya Ş., Cem E., Düzgöl M., Kara A.A., Arıkan K.Ö., Kantar A., Yılmaz E. (2022). Evaluation of Proximal Tubule Functions in Children with COVID-19: A Prospective Analytical Study. World J. Pediatr..

[5] Bjornstad E.C., Seifert M.E., Sanderson K., Feig D.I. (2022). Kidney Implications of SARS-CoV2 Infection in Children. Pediatr. Nephrol..

[6] Shah S.A., Carter H.P. (2020). New-Onset Nephrotic Syndrome in a Child Associated With COVID-19 Infection. Front. Pediatr..

[7] Basiratnia M., Derakhshan D., Yeganeh B.S., Derakhshan A. (2021). Acute Necrotizing Glomerulonephritis Associated with COVID-19 Infection: Report of Two Pediatric Cases. Pediatr. Nephrol..

[8] Stewart D.J., Mudalige N.L., Johnson M., Shroff R., du Pré P., Stojanovic J. (2022). Acute Kidney Injury in Paediatric Inflammatory Multisystem Syndrome Temporally Associated with SARS-CoV-2 (PIMS-TS) Is Not Associated with Progression to Chronic Kidney Disease. Arch. Dis. Child..

[9] Meneghel A., Masenello V., Alfier F., Giampetruzzi S., Sembenini C., Martini G., Tirelli F., Meneghesso D., Zulian F. (2023). Renal Involvement in Multisystem Inflammatory Syndrome in Children: Not Only Acute Kidney Injury. Children.

[10] Raina R., Mawby I., Chakraborty R., Sethi S.K., Mathur K., Mahesh S., Forbes M. (2022). Acute Kidney Injury in COVID-19 Pediatric Patients in North America: Analysis of the Virtual Pediatric Systems Data. PLoS ONE.

[11] Generalić A., Davidović M., Kos I., Vrljičak K., Lamot L. (2021). Hematuria as an Early Sign of Multisystem Inflammatory Syndrome in Children: A Case Report of a Boy With Multiple Comorbidities and Review of Literature. Front. Pediatr..

[12] Yousaf Z., Al-Shokri S.D., Al-Soub H., Mohamed M.F.H. (2020). COVID-19-Associated SIADH: A Clue in the Times of Pandemic!. Am. J. Physiol. Endocrinol. Metab..

[13] Mills T., Trivedi A., Tremoulet A.H., Hershey D., Burns J.C. (2021). Hyponatremia in Patients With Multisystem Inflammatory Syndrome in Children. Pediatr. Infect. Dis. J..

[14] Park S.J., Shin J.I. (2013). Inflammation and Hyponatremia: An Underrecognized Condition?. Korean J. Pediatr..

[15] Yoshimura M., Conway-Campbell B., Ueta Y. (2021). Arginine Vasopressin: Direct and Indirect Action on Metabolism. Peptides.

[16] Erdélyi L.S., Hunyady L., Balla A. (2023). V2 Vasopressin Receptor Mutations: Future Personalized Therapy Based on Individual Molecular Biology. Front. Endocrinol..

[17] Stockand J.D. (2010). Vasopressin Regulation of Renal Sodium Excretion. Kidney Int..

[18] Dalal N., Pfaff M., Silver L., Glater-Welt L., Sethna C., Singer P., Castellanos-Reyes L., Basalely A. (2023). The Prevalence and Outcomes of Hyponatremia in Children with COVID-19 and Multisystem Inflammatory Syndrome in Children (MIS-C). Front. Pediatr..

[19] Ludvigsson J.F. (2020). Systematic Review of COVID-19 in Children Shows Milder Cases and a Better Prognosis than Adults. Acta Paediatr..

[20] Biagetti B., Sánchez-Montalvá A., Puig-Perez A., Campos-Varela I., Pilia M.F., Anderssen-Nordahl E., González-Sans D., Miarons M., Simó R. (2024). Hyponatremia after COVID-19 Is Frequent in the First Year and Increases Re-Admissions. Sci. Rep..

[21] Meher B.K., Panda I., Sahoo J.P., Acharya G., Mohanty M., Naik S., Jena P.K., Mohakud N.K. (2023). Predictors of Mortality with Multisystem Inflammatory Syndrome in Children (MIS-C): A Single Centre Prospective Observational Study from Eastern India. J. Pediatr. Crit. Care.

[22] Nayak S., Panda P.C., Biswal B., Agarwalla S.K., Satapathy A.K., Jena P.K., Gulla K.M., Rath D., Mahapatra A., Mishra P. (2022). Eastern India Collaboration on Multisystem Inflammatory Syndrome in Children (EICOMISC): A Multicenter Observational Study of 134 Cases. Front. Pediatr..

[23] Basu S., Bhattacharyya A., Ray K., Ghosh A. (2020). Biomarker Profile in Pediatric Inflammatory Multisystem Syndrome Temporarily Associated with SARS-CoV-2 (PIMS-TS)/Multisystem Inflammatory Syndrome in Children (MIS-C). Indian J. Biochem. Biophys..

[24] Tran D.M., Pham D.V., Cao T.V., Hoang C.N., Nguyen H.T.T., Nguyen G.D., Le C.N., Thieu Q.Q., Ta T.A., Dau H.V. (2024). Severity Predictors for Multisystemic Inflammatory Syndrome in Children after SARS-CoV-2 Infection in Vietnam. Sci. Rep..

[25] Khidir R.J.Y., Ibrahim B.A.Y., Adam M.H.M., Hassan R.M.E., Fedail A.S.S., Abdulhamid R.O., Mohamed S.O.O. (2022). Prevalence and Outcomes of Hyponatremia among COVID-19 Patients: A Systematic Review and Meta-Analysis. Int. J. Health Sci..

[26] Petrea C.L., Ciortea D.A., Candussi I.L., Gurău G., Matei N.M., Bergheș S.E., Chirila S.I., Berbece S.I. (2024). A Study of Hydroelectrolytic and Acid–Base Disturbances in MIS-C Patients: A Perspective on Antidiuretic Hormone Secretion. Curr. Issues Mol. Biol..

[27] Petrone D., Mateo-Urdiales A., Sacco C., Riccardo F., Bella A., Ambrosio L., Lo Presti A., Di Martino A., Ceccarelli E., Del Manso M. (2023). Reduction of the Risk of Severe COVID-19 Due to Omicron Compared to Delta Variant in Italy (November 2021–February 2022). Int. J. Infect. Dis..

[28] Pinto Pereira S.M., Mensah A., Nugawela M.D., Stephenson T., Ladhani S.N., Dalrymple E., Dudley J., McOwat K., Simmons R., Heyman I. (2023). Long COVID in Children and Young after Infection or Reinfection with the Omicron Variant: A Prospective Observational Study. J. Pediatr..

[29] Saleh A.O., Al-Shokri S.D., Ahmed A.O., Musa A.E., Mohamed M.F. (2020). Urinary Retention and Severe Hyponatremia: An Unusual Presentation of COVID-19. Eur. J. Case Rep. Intern. Med..

[30] Pillai B.P., Unnikrishnan A.G., Pavithran P.V. (2011). Syndrome of Inappropriate Antidiuretic Hormone Secretion: Revisiting a Classical Endocrine Disorder. Indian J. Endocrinol. Metab..

